# Refractive outcomes of immediately sequential bilateral cataract surgery in eyes with long and short axial lengths

**DOI:** 10.1186/s12886-024-03347-3

**Published:** 2024-02-20

**Authors:** André S. Pollmann, Michael Trong Duc Nguyen, Milime Keyeutat, Éliane Danis, Georges M. Durr, Younes Agoumi, Samir Jabbour

**Affiliations:** 1 Department of Ophthalmology, Centre Hospitalier de l’Université de Montréal (CHUM), 1051 Sanguinet St, Montréal, Quebec, H2X 3E4 Canada; 2 Faculty of Medicine and Health Sciences, Université de Sherbrooke, Sherbrooke, QC Canada; 3 Department of Ophthalmology, McGill University, Montréal, Canada

**Keywords:** Immediately sequential bilateral cataract surgery, Axial length, Biometry, Hyperopia, Myopia

## Abstract

**Purpose:**

To report the refractive outcomes of long (≥25.00 mm) and short (≤22.00 mm) axial length (AL) eyes undergoing immediately sequential bilateral cataract surgery (ISBCS).

**Methods:**

In this retrospective cohort study, patients who underwent ISBCS were identified and eyes of patients with bilateral long and short ALs were included. Pre- and postoperative biometry, autorefraction, and ocular comorbidities or complications were recorded. The primary outcome was the mean refractive prediction error.

**Results:**

Thirty-seven patients (74 eyes) with long ALs and 18 patients (36 eyes) with short ALs were included. The means ± standard deviations of the ALs were 26.40 ± 1.38 mm and 21.44 ± 0.46 mm in the long and short AL groups, respectively. In long AL eyes, the mean absolute error from the biometry-predicted refraction was − 0.16 ± 0.46 D, corresponding to 74% of eyes achieving a refraction within ±0.50 D of the predicted value. In short AL eyes, the mean absolute error was − 0.63 ± 0.73 D, corresponding to 44% of eyes achieving a refraction within ±0.50 D of the predicted value. Eight (44.4%) patients with short AL eyes had a myopic deviation greater than ±0.50 D from the predicted result in both eyes.

**Conclusions:**

Compared to patients with long AL eyes, ISBCS in patients with short ALs had a wider variance in refractive outcome and a lower rate of achieving a postoperative refraction within ±0.50 D of the predicted target.

## Background

Age-related cataracts are usually bilateral; therefore, performing surgery on both eyes on the same day, termed immediately sequential bilateral cataract surgery (ISBCS), offers several logistical and economic advantages to patients and healthcare systems [1, 2]. Ophthalmologists have had concerns about ISBCS, especially postoperative refractive error in both eyes due to the inability to adjust the intraocular lens (IOL) power for the second surgery based on the refractive results of the first surgery [3, 4]. However, systematic comparisons of ISBCS and delayed sequential bilateral cataract surgeries (DSBCS) reveal minimal to no differences in refractive outcomes or complications [5, 6].

ISBCS has been suggested as a favorable option for patients with abnormally short or long eyes to avoid postoperative anisometropia [7]. However, biometry can be less accurate in eyes with abnormal ALs [8–10]. Short axial length (AL) eyes are considered to have an AL less than or equal to 22.00 mm [11, 12], while long AL eyes measure 25.00 mm or more [9, 13]. Furthermore, variations of up to 1.00 D are tolerated by manufacturers for IOLs with powers greater than 30 D, which can contribute to the risk of postoperative refractive surprise in short AL eyes [10]. Despite advances in biometry and IOL formulas, achieving target refraction remains a challenge for eyes with AL outside of the normal range. Indeed, up to 20% of eyes with an AL > 26.00 mm can experience postoperative deviation from their target refraction greater than ±0.50 D [14].

Few studies to date have assessed refractive outcomes in high myopic or hyperopic eyes undergoing ISBCS. Furthermore, the unavailability of axial length data [15, 16] and the exclusion of eyes with extremes of axial length [17, 18] are limitations of prior research. Therefore, this study examined the refractive outcomes of eyes with short or long AL undergoing ISBCS.

## Methods

This study retrospectively reviewed the electronic medical records of patients who underwent ISBCS for bilateral visually significant cataracts between August 1, 2020 and October 31, 2022 at the Centre Hospitalier de l’Université de Montréal, Montréal, Quebec, Canada. In this publicly funded hospital setting, ophthalmic nurses or technicians ascertained visual acuity and autorefraction at each patient visit. Any post-operative prescription of refractive correction and evaluation for laser refractive surgery was done in non-affiliated private healthcare settings.

Patients were eligible for inclusion if preoperative biometry showed bilateral AL ≤22.00 mm (short AL) or ≥ 25.00 mm (long AL). All types of implanted IOLs were considered, whether monofocal, extended depth of focus (EDOF), multifocal, or toric IOLs. Patients with prior corneal laser refractive surgery and ectasia were excluded, as were those missing postoperative follow-up data.

Ethics review board approval was obtained through the Centre hospitalier de l’Université de Montréal professional services department (PSD-21027), and the tenets of declaration of Helsinki were strictly adhered to while conducting the study.

### Surgical technique

Target refractions and IOLs were determined on an individual basis according to patient needs in discussion with the surgeon. IOL power was calculated with the Barrett Universal II formula. Surgeries were performed by an attending or resident in ophthalmology. Phacoemulsification techniques and IOL implantation into the capsular bag were performed as per the routine clinical practice of the ophthalmologist, all of which involved the use of topical anesthesia and a 2.2 mm clear-corneal main incision. Each eye was treated as a completely independent procedure.

### Data collection and follow-up

The preoperative variables extracted included patient demographics, ocular comorbidities, autorefraction (Nidek ARK-510A autorefractor, Nidek Co., Ltd.), biometric information (IOL Master700, Carl Zeiss Meditec AG), Snellen corrected visual acuity in each eye at 6 m, and surgeon-selected postoperative refractive targets. Follow-up examinations generally occurred on day 1, weeks 2–3, and weeks 6–8 postoperatively, or as per routine care by the individual surgeon. Postoperative outcomes, including autorefraction and uncorrected distance visual acuity, were extracted from the last available follow-up visit. Any intraoperative or postoperative complications were tabulated.

The primary outcome was the mean refractive absolute prediction error, defined as the difference between the postoperative observed refraction and the preoperative predicted refraction in the spectacle plane (in spherical equivalent, SE). The autorefraction in each eye at the final follow-up visit post-ISBCS was used as the observed refraction. Additional descriptive outcome measures included the proportion of eyes with a deviation from the target ±0.50 D and ± 1.00 D. Finally, we examined the percentage of patients who had a deviation greater than ±0.50 D in both eyes and in the same direction (either both myopic shifts or both hyperopic shifts), representing potential cases where the first-eye refractive result could have informed IOL selection in the second eye. The data were analyzed using Microsoft Excel 2019 software (Microsoft Corp., Redmond, WA).

## Results

A review of 1559 ISBCSs over 26 months identified 37 patients (74 eyes) with long ALs (≥25.00 mm) and 18 patients (36 eyes) with short ALs (≤22.00 mm) eligible for inclusion. Patients were between 55 and 85 years old (Table 1). In the long AL group, the ALs were between 25.00 mm and 27.00 mm in 53 eyes (71.6%), between 27.01 mm and 30.00 mm in 19 eyes (25.7%) and > 30.00 mm in 2 eyes (2.7%). In the short AL group, the AL was between 21.01 mm and 22.00 mm in 27 eyes (75.0%) and between 20.00 mm and 21.00 mm in 9 eyes (25.0%). No eyes had an AL < 20.00 mm.

**Table 1:** Pre-operative patient characteristics

	Axial length ≥ 25.00 mm *n* = 74 eyes (37 patients)	Axial length ≤ 22.00 mm *n* = 36 eyes (18 patients)
**Parameter**	**Mean ± SD**	
Age (y)	67.5 ± 7.7	69.9 ± 8.0
Male/Female (%)	48.6/51.4	11.0/89.0
Pre-operative best-corrected visual acuity, each eye (logMAR)	0.27 (Snellen 20/36) ± 0.28	0.37 (Snellen 20/45) ± 0.44
Corneal astigmatism on biometry (D)	0.95 ± 0.61	1.5 ± 1.2
Axial length (mm)	26.40 ± 1.38	21.44 ± 0.46
Pre-operative manifest refraction		
Sphere (D)	−6.78 ± 4.36	2.26 ± 2.42
Cylinder (D)	1.05 ± 1.05	1.11 ± 0.83
Spherical equivalent (D)	− 6.26 ± 4.07	2.82 ± 2.45
Implanted IOL power, sphere (D)	13.35 ± 3.78	29.08 ± 2.44
Biometry predicted spherical equivalent (D)	−0.34 ± 0.32	−0.39 ± 0.52

*IOL* Intraocular lens

Prior ocular diagnoses included dry eye disease (6 eyes in the long AL group), primary angle closure suspect (16 eyes in the short AL group), and open angle glaucoma or glaucoma suspect (14 eyes in the long AL group and 4 eyes in the short AL group). No intraoperative complications were noted, and all incisions were sealed without sutures.

In long AL eyes, the IOLs implanted were monofocal in 66 eyes (89.2%), including 8 monofocal toric IOLs (mean IOL cylinder 2.48 D, range 1.50 to 3.00 D); multifocal in 4 eyes (5.4%); and EDOF in 4 eyes (5.4%), including 2 toric EDOF IOLs. In all the short AL eyes, a monofocal IOL was implanted, 3 of which were toric IOLs (mean IOL cylinder 3.25 D, range 1.50 to 5.25 D).

### Prediction error for long AL eyes

**Fig. 1:**
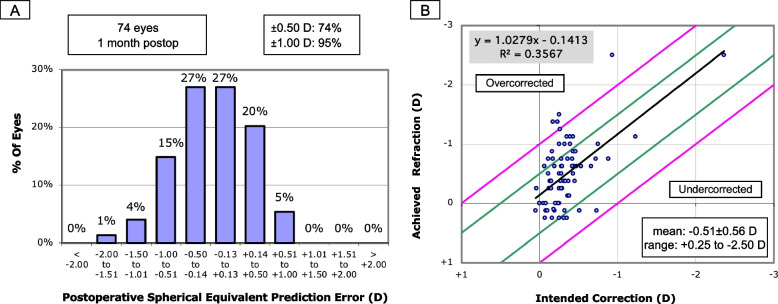
Histogram showing the distribution of postoperative spherical equivalent prediction errors (**a**) and scatter plot comparing predicted versus achieved spherical equivalent refraction (**b**) in 74 long axial length eyes (≥25.00 mm) at a median follow-up of 1 month after immediate sequential bilateral cataract surgery

Among long AL eyes, the SE autorefraction at last follow-up was myopic (defined as less than − 0.25 D) in 45 eyes (60.8%) and between + 0.25 D to − 0.25 D in 29 eyes (39.2%). The mean absolute error from the biometry-predicted postoperative refraction was − 0.16 ± 0.46 D (range − 1.57 D to + 0.86 D) (Table 2). The final refractions were within ±0.50 D and ± 1.00 D of the predicted result in 74.3 and 94.6% of eyes, respectively (Fig. 1a). The mean final SE refraction was − 0.51 ± 0.56 D (Fig. 1b). All eyes receiving multifocal or EDOF IOLs were within ±1.00 D of the predicted result. However, none of the eyes receiving multifocal IOLs and only two receiving a EDOF IOLs were within ±0.50 D of the biometry-predicted refraction post-operatively.

**Table 2:** Postoperative refractive outcomes

	Axial length ≥ 25.00 mm *n* = 74 eyes (36 patients)	Axial length ≤ 22.00 mm *n* = 36 eyes (18 patients)
**Parameter**	**Mean ± SD**	
Follow-up length (weeks)	6.26 ± 6.94	4.67 ± 3.85
Post-operative uncorrected distance visual acuity, each eye (logMAR)	0.10 (Snellen 20/25) ± 0.21	0.31 (Snellen 20/40) ± 0.43
Post-operative manifest autorefraction		
Sphere (D)	−0.86 ± 0.59	−1.50 ± 1.17
Cylinder (D)	0.71 ± 0.41	0.96 ± 1.27
Spherical equivalent (D)	−0.51 ± 0.56	−1.02 ± 0.95
Absolute predictive error (D)	−0.16 ± 0.46	−0.63 ± 0.73

Any bilateral SE hyperopic shift from the biometry-predicted result was observed in 11 (29.7%) long AL eyes (mean 0.31 ± 0.21 D). This resulted in 4 (11.1%) patients having bilateral low hyperopic SE on postoperative manifest refraction (mean SE + 0.17 D, range + 0.10 D to + 0.25 D). Bilateral deviations greater than ±0.50 D from the biometry-predicted result occurred in 5 patients (13.5%): one patient had a bilateral hyperopic shift (+ 0.86 D OD and + 0.64 D OS) and 4 had a bilateral myopic shift––including one patient who experienced a postoperative myopic surprise greater than 1.00 D in both eyes (− 1.15 D OD and − 1.25 D OS). Unilateral refractive surprises (defined as a final manifest SE refraction greater than ±1.00 D from the predicted SE) occurred in a total of 4 eyes (5.3%), all of which demonstrated a myopic shift.

### Prediction error for short AL eyes

**Fig. 2:**
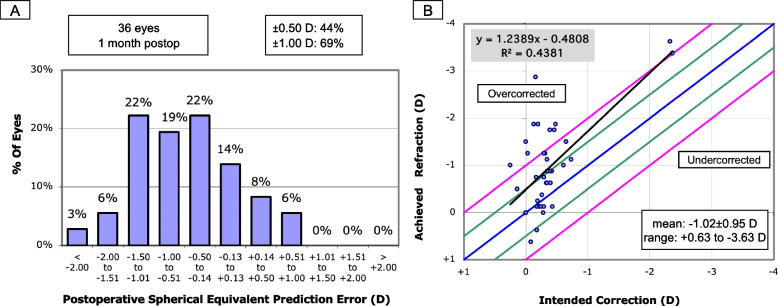
Histogram showing the distribution of postoperative spherical equivalent prediction errors (**a**) and scatter plot comparing predicted versus achieved spherical equivalent refraction (**b**) in 38 long axial length eyes (≤22.00 mm) at a median follow-up of 1 month after immediate sequential bilateral cataract surgery

Among the short AL eyes, the SE autorefraction at last follow-up was myopic (less than − 0.25 D) in 27 eyes (75.0%), hyperopic (greater than + 0.25 D) in 2 eyes (5.6%), and between + 0.25 D to − 0.25 D inclusive in 7 eyes (19.4%). The mean absolute error from the biometry-predicted postoperative refraction was − 0.63 ± 0.73 D (range − 2.73 D to + 0.71 D). The final refractions were within ±0.50 D and ± 1.00 D of the predicted result for 44.4 and 69.4% of eyes, respectively (Fig. 2a). The mean final SE refraction was − 1.02 ± 0.95 D (Fig. 2b).

A bilateral SE hyperopic shift from the biometry-predicted result was observed in 2 (11.1%) patients with short ALs (mean 0.46 ± 0.20 D). However, neither patient had a final hyperopic outcome in both eyes. Eight patients (44.4%) were found to have a myopic deviation greater than 0.50 D from the predicted result in both eyes. Four patients (22.2%) had myopic deviation greater than 1.00 D in both eyes. A refractive surprise greater than ±1.00 D SE from the biometry-predicted SE occurred in 11 eyes (30.6%), all of which demonstrated a myopic shift. These eyes were comparable to those within 1.00 D or less from the biometry-predicted outcome with regards to AL (21.32 ± 0.43 mm versus 21.49 ± 0.47 mm) and biometry-measured astigmatism (1.36 ± 0.95 D versus 1.56 ± 1.29 D).

### Complications

Post-operative transient ocular hypertension (intraocular pressure > 30 mmHg on Goldmann applanation tonometry) was reported in 9 (12.2%) long AL eyes (6 patients). Rebound uveitis occurred in 6 (8.1%) long AL eyes (3 patients) and 2 (5.6%) short AL eyes (1 patient). Rebound inflammation was attributed to poor corticosteroid drop adherence in one long AL patient. Cystoid macular edema and rhegmatogenous retinal detachment each occurred once, both in eyes with long ALs.

## Discussion

Although ISBCS has become increasingly popular, few data exist regarding refractive outcomes in patients with bilateral short or long AL. AL is a critical variable in IOL calculations; a 1 mm error in AL measurement translates to an over 2 D error in IOL power in an average eye of approximately 23.7 ± 1.2 mm and may translate to far greater refractive errors in shorter AL eyes [19, 20]. The purpose of this study was to report the refractive outcome data of ISBCSs in patients with AL ≤22.00 mm or ≥ 25.00 mm. We found that patients with bilateral short AL have higher rates of refractive surprise in both eyes compared to patients with bilateral long ALs.

With advances in IOL formulas, 70–90% of eyes are expected to achieve a refraction within ±0.50 D of predicted after cataract surgery with posterior chamber lens implantation [9, 16, 21], a target that is proposed as the threshold for spectacle independence [22]. The Barrett Universal II formula used in our study is a fourth-generation formula that appears to have a low bias as measured by prediction error with variations in AL, making it a reasonable choice for calculating IOL power in both long and short eyes [9, 23]. In our long AL eyes, the postoperative refraction was ±0.50 D of predicted for 74% of the eyes and within ±1.00 D for 95% of the eyes. However, in short AL eyes, the postoperative refraction was within ±0.50 D of the predicted result in only 44% of eyes and within ±1.00 D in 69% of eyes. These findings match other studies examining single eye outcomes [13, 24]. In the context of ISBCS where there is no opportunity for IOL adjustment between eyes, surgeons may consider informing short AL patients of a higher incidence of unpredictable refractive outcome.

For DSBCS, correction methods aimed at improving the outcome in the second eye after a refractive surprise in the first eye include accounting for 50% of the first eye error in the selection of the IOL in the second eye [25] or applying a regression analysis unique to the IOL formula [26]. Yet studies analyzing the refractive outcomes of ISBCS suggest that adjusting the IOL power for the second eye based on the refractive outcome is not justified in most cases [27–29]. For example, in a retrospective study of 110 patients (220 eyes), Guber et al. reported achieving within ±0.50 D SE of target refraction in 60% of eyes and deemed only 5% of patients potentially benefited from IOL adjustment in the second eye [30]. Notably, abnormal AL has been an exclusion criterion in some prior studies of ISBCS [17, 18] or AL data have been unavailable for analysis [15, 16]. Given the less predictable refractive outcomes following ISBCS in patients with abnormal ALs (especially ≤22.00 mm), these patients should be distinguished from the regular ALs, which experience comparable outcomes to ISBCS. Our finding that 44% of patients with bilateral short ALs had postoperative refractions greater than ±0.50 D from the predicted value in both eyes, suggests that DSBCS with potential IOL power adjustment based on the refractive result in the first eye should be further studied in these eyes.

This study has several limitations. First, the use of autorefraction as a method for determining refractive error contrasts with the gold standard of subjective manifest refraction [31], although some studies support its use in evaluating post-cataract surgery refractive outcomes [32, 33]. Notably, it has been reported that autorefraction after multifocal IOL implantation may result in more negative SE values than subjective refraction [34, 35]. Second, the lens choice was not homogenous in our long AL cohort, as four eyes received EDOF IOLs and four eyes received multifocal IOLs. Given the small number of eyes, these were not analyzed separately. The use of these IOLs is an important consideration in long and short AL eyes undergoing ISBCS, as patients choosing these IOLs may have higher expectations of achieving post-operative spectacle independence. For multifocal IOLs, a range of IOL formulas, including the Barrett Universal II, were shown to achieve within ±0.50 D of the predicted refraction in 80–84% of eyes, especially in a small subset with AL longer than 26.00 mm [36]. However, none of the four eyes receiving a multifocal IOL in our study were within ±0.50 D of the predicted refraction. Third, the patients’ subjective perspectives were not captured, and it is possible that the refractive errors reported in our study do not correspond to meaningful clinical differences or degree of satisfaction. Fourth, the use of only the Barrett Universal II formula in this study means that the findings are not necessarily generalizable to newer IOL formulas. Although a widely studied topic, there remains uncertainty about the optimal formula in long and short AL eyes. Recently, the newer Olsen and Kane formulas have been suggested to deliver better accuracy compared to other formulas in both long and short AL eyes [21, 37].

## Conclusions

To the best of our knowledge, this study is the first to report the outcomes of ISBCSs in patients with long and short ALs. Using the Barrett Universal II formula, this study revealed that ISBCSs in eyes with an AL ≥25.00 mm achieve postoperative refractive targets at comparable rates to those in other reports in the literature. However, the eyes of patients with bilateral ALs ≤22.00 mm achieved lower refractive accuracy and experienced a wider range of SE errors. Clinically, patients with bilateral short AL eyes represent a unique population for which the average outcomes of larger studies of ICBCS may not necessarily apply. Although AL should be considered when counseling patients preoperatively, further studies validating these findings are warranted, as are studies examining approaches to IOL adjustment strategies for DSBCS in patients with bilateral short ALs.

## Data Availability

The raw dataset is not publicly available to respect the personal information of individuals’ as per Quebec legislation. The data that support the findings of this study are available from the corresponding author upon reasonable request.

## Abbreviations

AL: Axial length
DSBCS: Delayed sequential bilateral cataract surgeries
EDOF: Extended depth of focus
IOL: Intraocular lens
ISBCS: Immediately sequential bilateral cataract surgery
SE: Spherical equivalent

## References

[1] Miller KM, Oetting TA, Tweeten JP, Carter K, Lee BS, Lin S (2022). Cataract in the adult eye preferred practice pattern. Ophthalmology..

[2] Campbell CG, La CJ, Chan KL, Turnbull AMJ (2023). Patient satisfaction and attitudes towards immediate sequential bilateral cataract surgery. Eur J Ophthalmol.

[3] Grzybowski A, Wasinska-Borowiec W, Claoué C (2016). Pros and cons of immediately sequential bilateral cataract surgery (ISBCS). Saudi J Ophthalmol.

[4] You E, Hébert M, Arsenault R, Légaré MÈ, Mercier M. Perception of Canadian ophthalmologists on immediately sequential bilateral cataract surgery: insights and implications. Can J Ophthalmol. 2023; 10.1016/j.jcjo.2023.04.012. 10.1016/j.jcjo.2023.04.012 37290485

[5] Dickman MM, Spekreijse LS, Winkens B, Schouten JS, Simons RW, Dirksen CD (2022). Immediate sequential bilateral surgery versus delayed sequential bilateral surgery for cataracts. Cochrane Database Syst Rev.

[6] Friling E, Johansson B, Lundström M, Montan P (2022). Postoperative endophthalmitis in immediate sequential bilateral cataract surgery: a nationwide registry study. Ophthalmology..

[7] Kontkanen M, Kaipiainen S (2002). Simultaneous bilateral cataract extraction: a positive view. J Cataract Refract Surg.

[8] Kane JX, Melles RB (2020). Intraocular lens formula comparison in axial hyperopia with a high-power intraocular lens of 30 or more diopters. J Cataract Refract Surg.

[9] Melles RB, Holladay JT, Chang WJ (2018). Accuracy of intraocular lens calculation formulas. Ophthalmology..

[10] Hoffman RS, Vasavada AR, Allen QB, Snyder ME, Devgan U, Braga-Mele R (2015). Cataract surgery in the small eye. J Cataract Refract Surg.

[11] Hoffer KJ (1993). The Hoffer Q formula: a comparison of theoretic and regression formulas. J Cataract Refract Surg.

[12] Wang Q, Jiang W, Lin T, Wu X, Lin H, Chen W (2018). Meta-analysis of accuracy of intraocular lens power calculation formulas in short eyes. Clin Experiment Ophthalmol.

[13] Röggla V, Langenbucher A, Leydolt C, Schartmüller D, Schwarzenbacher L (2021). Accuracy of common IOL power formulas in 611 eyes based on axial length and corneal power ranges. Br J Ophthalmol.

[14] Voytsekhivskyy OV, Hoffer KJ, Tutchenko L, Cooke DL, Savini G (2023). Accuracy of 24 IOL power calculation methods. J Refract Surg.

[15] Owen JP, Blazes M, Lacy M, Yanagihara RT, Van Gelder RN, Lee AY (2021). Refractive outcomes after immediate sequential vs delayed sequential bilateral cataract surgery. JAMA Ophthalmol.

[16] Lundström M, Dickman M, Henry Y, Manning S, Rosen P, Tassignon MJ (2018). Risk factors for refractive error after cataract surgery: analysis of 282 811 cataract extractions reported to the European registry of quality outcomes for cataract and refractive surgery. J Cataract Refract Surg.

[17] Serrano-Aguilar P, Ramallo-Fariña Y, Cabrera-Hernández JM, Perez-Silguero D, Perez-Silguero MA, Henríquez-de la Fe F (2012). Immediately sequential versus delayed sequential bilateral cataract surgery: safety and effectiveness. J Cataract Refract Surg.

[18] Sarikkola AU, Uusitalo RJ, Hellstedt T, Ess SL, Leivo T, Kivelä T (2011). Simultaneous bilateral versus sequential bilateral cataract surgery: Helsinki simultaneous bilateral cataract surgery study report 1. J Cataract Refract Surg.

[19] Lee KE, Klein BE, Klein R, Quandt Z, Wong TY (2009). Association of age, stature, and education with ocular dimensions in an older white population. Arch Ophthalmol.

[20] Gavin EA, Hammond CJ (2008). Intraocular lens power calculation in short eyes. Eye (Lond).

[21] Kane JX, Chang DF (2021). Intraocular lens power formulas, biometry, and intraoperative aberrometry: a review. Ophthalmology..

[22] Villegas EA, Alcón E, Artal P (2014). Minimum amount of astigmatism that should be corrected. J Cataract Refract Surg.

[23] Kane JX, Van Heerden A, Atik A, Petsoglou C (2016). Intraocular lens power formula accuracy: comparison of 7 formulas. J Cataract Refract Surg.

[24] Shrivastava AK, Behera P, Kumar B, Nanda S (2018). Precision of intraocular lens power prediction in eyes shorter than 22 mm: an analysis of 6 formulas. J Cataract Refract Surg.

[25] Covert DJ, Henry CR, Koenig SB (2010). Intraocular lens power selection in the second eye of patients undergoing bilateral, sequential cataract extraction. Ophthalmology..

[26] Olsen T (2011). Use of fellow eye data in the calculation of intraocular lens power for the second eye. Ophthalmology..

[27] Jabbour J, Irwig L, Macaskill P, Hennessy MP (2006). Intraocular lens power in bilateral cataract surgery: whether adjusting for error of predicted refraction in the first eye improves prediction in the second eye. J Cataract Refract Surg.

[28] Herrinton LJ, Liu L, Alexeeff S, Carolan J, Shorstein NH (2017). Immediate sequential vs. delayed sequential bilateral cataract surgery: retrospective comparison of postoperative visual outcomes. Ophthalmology..

[29] Spekreijse L, Simons R, Winkens B, van den Biggelaar F, Dirksen C, Bartels M (2023). Safety, effectiveness, and cost-effectiveness of immediate versus delayed sequential bilateral cataract surgery in the Netherlands (BICAT-NL study): a multicentre, non-inferiority, randomised controlled trial. Lancet..

[30] Guber I, Rémont L, Bergin C (2015). Predictability of refraction following immediate sequential bilateral cataract surgery (ISBCS) performed under general anaesthesia. Eye Vis (Lond).

[31] Kemchoknatee P, Sunlakaviset P, Khieokhoen N, Srisombut T, Tangon D (2023). A comparison of autorefraction and subjective refraction in an academic optometry clinic. Cureus..

[32] Bissen-Miyajima H, Minami K, Yoshino M, Nishimura M, Oki S (2010). Autorefraction after implantation of diffractive multifocal intraocular lenses. J Cataract Refract Surg.

[33] Or L, Jacques A, Barrett GD (2022). Autorefraction as an objective method to evaluate accuracy of intraocular lens calculation formulas. J Refract Surg.

[34] Muñoz G, Albarrán-Diego C, Sakla HF (2007). Validity of autorefraction after cataract surgery with multifocal ReZoom intraocular lens implantation. J Cataract Refract Surg.

[35] Garzón N, García-Montero M, López-Artero E, Poyales F, Albarrán-Diego C (2019). Influence of trifocal intraocular lenses on standard autorefraction and aberrometer-based autorefraction. J Cataract Refract Surg.

[36] Lwowski C, Kohnen T (2023). Prospective evaluation of the ESCRS online calculator for calculation of a multifocal intraocular lens. J Cataract Refract Surg.

[37] Ma Y, Xiong R, Liu Z, Young CA, Wu Y, Zheng D (2024). Network meta-analysis of intraocular lens power calculation formula accuracy in 1016 eyes with long axial length. Am J Ophthalmol.

